# Accuracy of Combined High Tibial Slope Correction Osteotomy Using 3-Dimensional-Planned Patient-Specific Instrumentation

**DOI:** 10.1177/03635465241295726

**Published:** 2024-11-24

**Authors:** Christoph Zindel, Sandro Hodel, Lukas Jud, Stefan M. Zimmermann, Lazaros Vlachopoulos, Sandro F. Fucentese

**Affiliations:** † Department of Orthopaedics, Balgrist University Hospital, University of Zurich, Zurich, Switzerland; Investigation performed at the Department of Orthopaedics, Balgrist University Hospital, University of Zurich, Zurich, Switzerland

**Keywords:** high tibial osteotomy, posterior slope, patient-specific instruments, 3D preoperative planning.

## Abstract

**Background:**

If an increased posterior tibial slope (PTS) and concomitant unicompartmental osteoarthritis are present, a simultaneous sagittal (slope) and coronal correcting high tibial osteotomy has been recommended. However, no study has investigated the accuracy of such combined high tibial slope correction osteotomies.

**Purpose:**

(1) To report the accuracy of navigated high tibial slope correction osteotomies using patient-specific instruments (PSI) and (2) to analyze the influence of an open wedge osteotomy (OWO) versus a closed wedge osteotomy (CWO) and the hinge axis angle (HAA) on the accuracy of the PTS correction.

**Study Design:**

Cohort study; Level of evidence, 3.

**Methods:**

All PSI PTS-reducing osteotomies performed at 1 institution between 2019 and 2022 were reviewed. Three-dimensional (3D) accuracy was defined as the mean absolute 3D angular difference between the planned and achieved surgical correction (in degrees) in 3D models of computed tomography data. The influence of OWO versus CWO and the HAA on the reported accuracy was analyzed and a cutoff defined using receiver operating characteristic curve analysis.

**Results:**

Eighteen patients who underwent a slope-reducing CWO (n = 9) or OWO (n = 9) were included. The 3D accuracy for PTS was 2.3°± 1.1° (mean ± SD), with CWO being more accurate than OWO (1.4°± 0.9° vs 3.1°± 0.6°; *P* < .01). Accuracy strongly correlated with the HAA (*r* = 0.788; *P* < .01). An HAA >38.9° predicted a PTS error >2° (odds ratio, 1.12 [95% CI, 1.04-1.20; *P* = .004]; area under the curve, 0.95 [95% CI, 0.89-1.00; *P* < .001]) corresponding to a coronal/sagittal correction of 0.8:1.

**Conclusion:**

Slope-reducing osteotomy can accurately be achieved using PSI. CWO demonstrated an increased accuracy when compared with OWO, which strongly depended on the HAA. With an aim of combined PTS and coronal correction, CWO should be considered the primary choice for accurate slope reduction with a coronal/sagittal correction cutoff of 0.8:1 (HAA, 38.9°).

It is well established that an increased posterior tibial slope (PTS) is a risk factor for anterior cruciate ligament (ACL) injuries^12^ and graft failure.^17^ Hence, in knees with ACL deficiency and increased PTS, a slope-reducing osteotomy should be considered.^7,43^ Imhoff et al^24^ showed that in such chronic cases of ACL deficiency, concomitant unicompartmental osteoarthritis is frequently present. Therefore, simultaneous sagittal (slope) and coronal correction would be preferable.

In clinical practice, the standard high tibial osteotomy (HTO) is still predominantly performed using the conventional freehand technique^29^ based on preoperative 2-dimensional (2D) planning. However, relying solely on 2D images can neither sufficiently delineate 3-dimensional (3D) changes of the anatomy nor allow accurate planning.^26,28^ This limitation may lead to inferior operation outcomes.^14^ Furthermore, the intraoperative use of patient-specific instruments (PSIs) allows a precise execution of 3D surgical planning, which has been demonstrated for different types of osteotomies.^31,46,48^ Jacquet et al^27^ showed that navigated HTO shortens the operating time as well as the learning curve and reduces the use of fluoroscopy time. During the planning and performing of an osteotomy for simultaneous correction of PTS and coronal deformity (so called combined high tibial slope correction osteotomies (HTSCOs)), the hinge axis may be rotated on the cutting plane according to the ratio of the combined deformity. This can lead to extreme hinge axis positions as described by Eliasberg et al^11^ and Imhoff and Vlachopoulos.^25^ HTSCOs are technically challenging, and 3D planning and PSI might even play a bigger role than in the standard coronal HTO. Nevertheless, there has been no study investigating the accuracy of HTSCOs using PSI.

A variety of PTS correction techniques exist.^2,6-8,18^ They can mainly be grouped into opening wedge osteotomies (OWOs) and closing wedge osteotomies (CWOs). Several studies have compared the influence of these groups on clinical outcome and survivorship without clear superiority of one technique over the other.^1,4,33^ However, their influence on 3D accuracy remains largely unstudied.

Therefore, the aim of the present study was (1) to evaluate the accuracy of PSI-navigated HTSCO and (2) to analyze the influence of OWO, CWO, and the hinge axis angle (HAA) on the accuracy of the PTS correction. We hypothesized (1) that the accuracy of PSI-navigated HTSCO will be high and (2) that CWO will be more accurate than OWO for extreme hinge axis positions, especially if the sagittal correction exceeds the coronal correction.

## Methods

The study was approved by the local ethical committee (Zurich Cantonal Ethics Commission, PB_ 2023-00389), and all patients gave their informed consent.

### Study Cohort

All patients who underwent an HTSCO using PSI at our institution between January 2019 and July 2022 were reviewed (n = 27). Exclusion criteria were PTS-increasing operations (n = 2), dome osteotomy (n = 1), or missing postoperative computed tomography (CT) scans (n = 6). This led to 18 included patients.

### 3D Measurements and Surgical Planning

Preoperative planning included standing long leg radiographs, radiographs of the knee, and CT scan of the lower extremities. The CT data permitted image segmentation to reconstruct 3D triangular surface models, as previously described in literature.^23,34,36,37,49,50^ Further 3D planning was performed similar to the published procedure of Fucentese et al^14^ after postprocessing the segmented 3D surface models using in-house 3D planning software (CASPA; Balgrist CARD AG).

A computer algorithm was used to analyze the hip-knee-ankle angle (including 3D measurements for valgus) and to measure the 3D slope.^15^ A 3D coordinate system was defined according to Grood and Suntay,^16^ with the z-axis equal to the directional vector of the anatomic tibia axis, defined by an oriented bounding box.^47^ The x-axis represented the lateral direction, and the y-axis denoted the anterior direction. To define the mechanical axis of the femur and tibia and to measure the 3D hip-knee-ankle angle, the method described by Roth et al^41^ was used. The 3D valgus was then measured in the coronal plane as the projected 2D angle between the mechanical axis of the femur and tibia. To measure the 3D slope, the algorithm first defined the joint line plane as the average plane of 10 surface registration points on the medial and lateral tibial plateau in a standardized fashion as described by Hodel et al.^22^ The 3D slope was then measured in the sagittal plane as the projected 2D angle between the joint line plane and the mechanical axis of the tibia.

Sagittal correction (of the PTS) was planned to reach the nominal PTS value^40^ of 5° but was individualized by the underlying pathology. Coronal correction was individually tailored per the degree of degeneration while avoiding an oblique joint line (mechanical medial proximal tibial angle (mMPTA) >95°), similar to the description by Feucht et al.^13^ The planning of CWO was preferred over OWO if the main correction was in the sagittal plane (slope) rather than in the coronal, ultimately depending on the surgeon's choice. The aforementioned computer algorithm^15^ was then used to predict the orientation of the correction plane as well as the orientation of the hinge axis for the desired combined sagittal and coronal correction.^25^ As a basis to determine the exact location of the osteotomy planes and hinge axis, documentation by DePuy Synthes^9^ was used for the TomoFix Medial High Tibial Plate surgical technique. Therefore, the hinge axis was placed on the osteotomy plane 5 mm central to the cortical surface of the tibia and then oriented on the axial plane according to the ratio between the desired sagittal and desired coronal correction. All CWOs were planned with an infratuberosity osteotomy. For the OWO, the choice between infra- or supratuberosity osteotomy depended on the amount of correction and concomitant pathologies of the patellofemoral joint.^23^ The final planning was then assessed by the treating surgeon. It was adjusted considering the measured mechanical leg axis on the standing long leg radiographs (ie, in the weightbearing condition), the surgical approach, and again the symptoms of the patient. The final step of the preoperative planning was the positioning of the TomoFix Medial High Tibial Plate and the implant screws in 3D.

### Surgical Procedure

**Figure 1.**
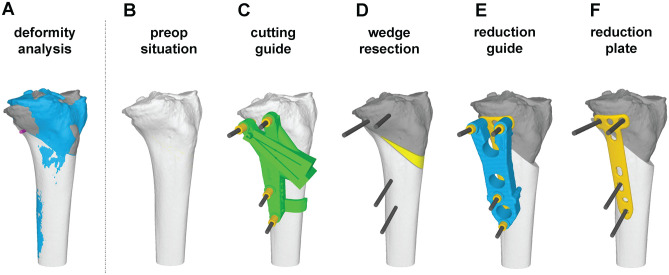
Surgical planning and procedure. (A) Three-dimensional bone model of the pathologic bone (blue) and the planned postoperative situation (dark gray: proximal part; light gray: distal part). (B) Three-dimensional bone model of the pathologic bone. (C) Patient-specific instruments (guide) for cutting. (D) Performed cuts and resected wedge (yellow). (E) Reduction and application of reduction guide. (F) Fixation of reduction with TomoFix plate.

All surgical procedures were performed according to the description by Fucentese et al.^14^ They were performed using general or spinal anesthesia, with the patients in the supine position and with an applied tourniquet. An anteromedial approach to the tibial head was performed. The bone was detached from the soft tissue to reveal the predefined bony landmarks, which had been identified as being relevant for proper positioning and were therefore integrated in the undersurface of all the used PSIs (guides). First, the cutting guide was positioned and fixed with reference Kirschner wires (K-wires). The position of the K-wires was planned in the postoperative screw positions of the used implant (TomoFix Medial High Tibial Plate). The use of sufficiently large K-wires (4 mm) eliminated the need for predrilling for the subsequent screws (5 mm) in later steps of the procedure. The cutting guide served as a registration tool between the 3D preoperative planning and the intraoperative situation. The reference K-wires served as orientation for the following guides. While the cutting guide was applied, full contact to the bone surface needed to be ensured and verified visually. Afterward, the osteotomy was performed. The cutting guide defined the position, orientation, and cutting depth of the preplanned osteotomy plane. Subsequently, the reduction guide was placed. This facilitated exact predefined reduction according to the reference K-wires, and it permitted placement and fixation of the TomoFix Medial High Tibial Plate over the preplanned screw holes. To confirm the osteotomy, reduction, and positioning of the implant, fluoroscopy was used. Figure 1 highlights the main steps of the surgical planning and procedure.

### Aftercare

A standard aftercare protocol of our institution was applied for all cases. It included partial weightbearing with 15 kg on the first day after the procedure for 6 weeks. Clinical follow-up was arranged at 6 and 18 weeks as well as 1 year after the procedure. Plain radiographs of the knee were acquired on the second postoperative day and all the appointments for clinical follow-up, whereas standing long leg radiographs were acquired only at the appointments for clinical follow-up at 18 weeks and 1 year after the procedure. To confirm bony consolidation, CT scans were obtained at the second clinical follow-up at 18 weeks postoperatively.

### Accuracy Analysis

**Figure 2.**
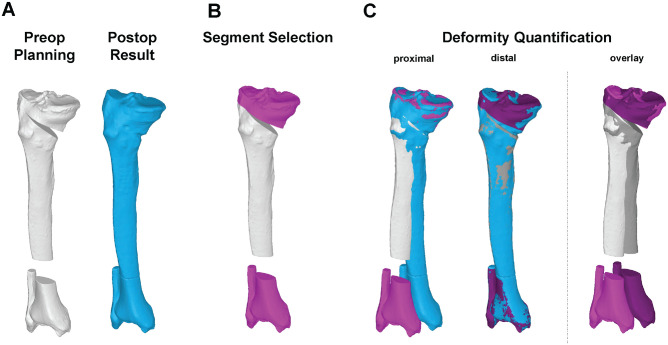
Three-dimensional (3D) accuracy analysis. (A) A 3D bone model of the preoperative planning (white) is paired with the 3D bone model of the postoperative result (blue). (B) Proximal and distal segments (magenta) are selected. (C) Both segments are superimposed with the postoperative result. The 3D rotational and translational difference between these segments quantifies the accuracy.

3D accuracy was calculated as the difference between planned and achieved correction^20,21,42,49^ by superimposing the corresponding 3D models of the proximal and distal tibia accordingly (Figure 2). Accuracy was reported as an absolute value (positive value without regard to its sign). The 3D accuracy in rotation (degrees) and translation (millimeters) was measured with respect to the coronal, axial, and sagittal planes as well as independent of the coordinate system as transformation shift and transformation angle. The HAA was measured in the preoperative planning in the axial plane with respect to the coronal plane.^11^ The HAA has a value between 0° (hinge parallel to coronal plane) and 90° (hinge axis parallel to sagittal axis).

### Statistical Analysis

All 3D measurements were expressed by mean, standard deviation, and range. All parameters were tested with the Shapiro-Wilk test for normality, and a 2-tailed unpaired *t* test or Mann-Whitney test was applied as appropriate. To compare differences in the demographics, a chi-square test was performed. A Pearson correlation was performed to measure the relation between the accuracy and the HAA. To identify a cutoff of the HAA that leads to a decreased accuracy of >2° of slope correction, receiver operating characteristic curve analysis and binary logistic regression were performed.

Given the highly standardized and largely automated measurement procedure, no inter- or intrareader reliability was performed.^19,30^ PTS was described as positive values, while negative values represented anterior tibial slope. Valgus deformities were described as positive values and varus deformities as negative values. *P* values <.05 were considered statistically significant. Statistical analysis was performed with SPSS Statistics for Windows (Version 26.0; IBM).

## Results

The study included 18 patients (16 male, 2 female) with a mean age of 31.1 ± 10.4 years (range, 17-47) at the time of surgery. All demographics are summarized in Table 1. The indication for the PTS correction included chronic ACL deficiency with an increased PTS (n = 15), extension deficit (n = 1), osteoarthritis with an increased PTS of 15° (n = 1), or revision after HTO with an unintentional increase of PTS (n = 1). The indication for the additional coronal leg axis correction included patients with medial osteoarthritis grades 1 to 3 (Kellgren and Lawrence classification^32^; n = 15) or chronic ACL deficiency with an increased valgus (n = 3).

**Table 1:** Patient Characteristics^*a*^

	CWO (n = 9)	OWO (n = 9)	Total (N = 18)	*P*-Value
Sex, No. (%)				
Male	8 (89)	8 (89)	16 (89)	≥.999^*b*^
Female	1 (11)	1 (11)	2 (11)	≥.999^*b*^
Age, y				.005^*c*,*d*^
Mean	24.9	37.2	31.1	
SD	7.2	9.4	10.4	
Range	17-37	23-47	17-47	
Body mass index				.124^*c*^
Mean	24.1	26.5	25.3	
SD	4.8	3.1	4.2	
Range	17.6-33.4	22.3-31.4	17.6-33.5	
Side, No. (%)				
Right	8 (89)	7 (78)	15 (83)	.796^*b*^
Left	1 (11)	2 (22)	3 (17)	.564^*b*^

a CWO, closed wedge osteotomy; OWO, open wedge osteotomy.

b Chi-square test.

c *T* test.

d Statistically significant at *P* < .05.

Mean PTS was corrected from a mean of 9.1°± 3.4° to 4.8°± 4.2° in all patients (*P* < .01) by either OWO (n = 9) or CWO (n = 9). Concomitant coronal deformity was corrected from −2.3°± 3.4° to 0.0°± 3.0° (*P* = .02). The preoperative 3D deformity analysis showed no significant difference between the groups (Table 2).

**Table 2:** Pre- and Postoperative 3-Dimensional Measurements^*a*^

Deformity Analysis	CWO (n = 9)	OWO (n = 9)	Total (N = 18)	*P*-Value^*b*^
Preoperative				
PTS, deg				.228
Mean	8.4	9.7	9.1	
SD	4.1	2.2	3.4	
Range	3.7 to 17.4	6.1 to 15.0	3.7 to 17.4	
Coronal/valgus, deg				.060
Mean	−1.0	−3.6	−2.3	
SD	3.0	3.3	3.4	
Range	−8.0 to 2.4	−9.8 to 3.2	−9.8 to 3.2	
Postoperative: achieved correction				
PTS, deg				<.001^*c*^
Mean	1.9	7.8	4.8	
SD	3.1	3.0	4.2	
Range	−1.4 to 7.9	3.4 to 15.3	−1.4 to 15.3	
Coronal/valgus, deg				.044^*c*^
Mean	−1.2	1.3	0.0	
SD	2.4	3.0	3.0	
Range	−6.7 to 1.2	−2.8 to 8.5	−6.7 to 8.5	

a Valgus deformities are described as positive values, whereas varus deformities are described as negative values. CWO, closed wedge osteotomy; OWO, open wedge osteotomy; PTS, posterior slope.

b Unpaired *t* test.

c Statistically significant at *P* < .05.

### 3D Accuracy Analysis

**Figure 3.**
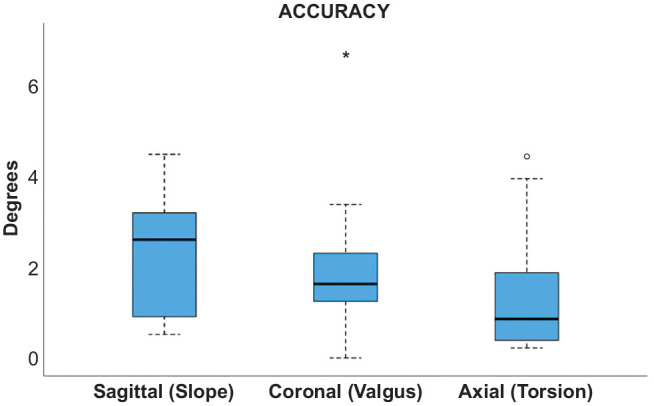
Three-dimensional accuracy. The box plot shows the mean and interquartile range (IQR) of the 3-dimensional accuracy of all 18 cases. Line, median; box, IQR; error bars, 1.5 x IQR; circle and asterix, outliners.

**Figure 4.**
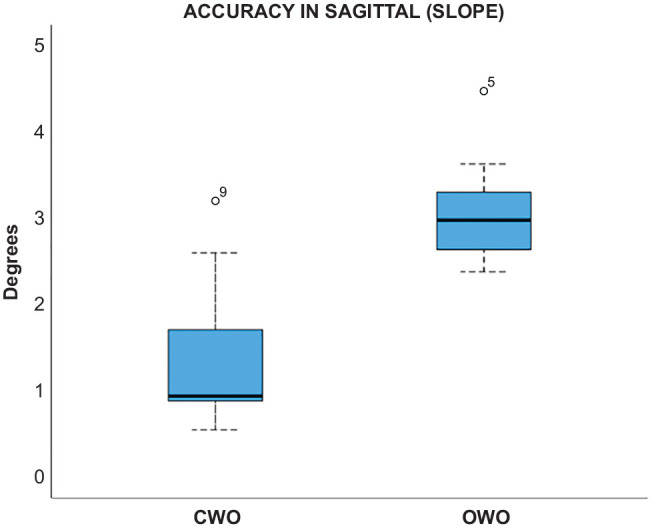
3-Dimensional accuracy in the sagittal plane showing difference between closed wedge osteotomy (CWO) and open wedge osteotomy (OWO). Line, median; box, interquartile range (IQR); error bars, 1.5 x IQR; circle, outliners.

3D accuracy was 2.3°± 1.1° for PTS (sagittal plane), 1.9°± 1.4° for valgus (coronal plane), and 1.4°± 1.2° in the axial plane (Figure 3). CWO was more accurate than OWO in PTS correction (1.4°± 0.9° vs 3.1°± 0.6°; *P* < .01) (Figure 4). All accuracy measurements are summarized in Table 3.

**Table 3:** Accuracy Measurement Between Planned and Achieved Reduction^*a*^

Accuracy	CWO (n = 9)	OWO (n = 9)	Total (N = 18)	*P*-Value^*b*^
Rotational 3-dimensional				
Sagittal/PTS, deg				<.001^*c*^
Mean	1.4	3.1	2.3	
SD	0.9	0.6	1.1	
Coronal/valgus, deg				.471
Mean	2.0	1.9	1.9	
SD	1.7	1.0	1.4	
Axial, deg				.385
Mean	1.5	1.3	1.4	
SD	1.5	0.7	1.2	
Translational 3-dimensional				
Sagittal, mm				.376
Mean	2.1	2.4	2.2	
SD	2.4	1.4	2.0	
Coronal, mm				.253
Mean	1.8	2.4	2.1	
SD	1.8	1.8	1.8	
Axial, mm				.416
Mean	2.1	2.3	2.2	
SD	1.6	1.4	1.5	
Coordinate independent				
TFS				.023^*c*^
Mean	2.5	1.7	2.1	
SD	0.8	0.7	0.9	
TFA				.182
Mean	3.2	4.0	3.6	
SD	2.2	0.8	1.7	

a All data are reported as absolutes values. CWO, closed wedge osteotomy; OWO, open wedge osteotomy; PTS, posterior slope; TFA, transformation angle; TFS, transformation shift.

b Unpaired *t* test.

c Statistically significant at *P* < .05.

**Figure 5.**
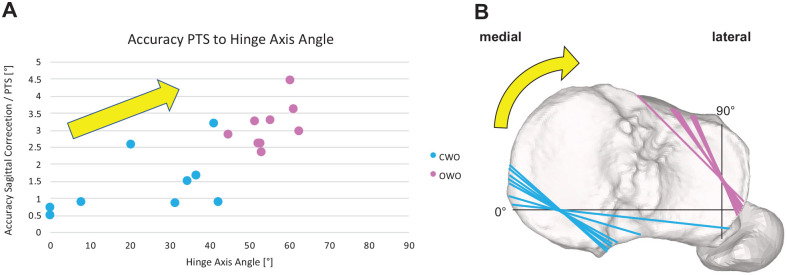
Accuracy of PTS related to HAA. (A) Strong correlation of PTS accuracy to HAA. CWO, light blue; OWO, purple. (B) All hinge axes (CWO in light blue, OWO in purple) projected onto a right tibia. The black axes marked 0° and 90° represent the HAA coordinate system. The yellow arrows indicate the increasing HAA and therefore the relationship of the 2 diagrams. CWO, closed wedge osteotomy; HAA, hinge axis angle; OWO, open wedge osteotomy; PTS, posterior tibial slope.

The HAA in CWO was significantly more parallel to the coronal plane as compared with OWO (23.7°± 16.2° vs 54.6°± 5.3°; *P* < .01). The accuracy of the slope correction strongly correlated with the HAA (*r* = 0.788; *P* < .01), as seen in Figure 5. A HAA cutoff of 38.9° predicted a slope accuracy of <2° with an odds ratio of 1.12 (95% CI, 1.04-1.20; *P* = .004), a sensitivity of 91.7%, a specificity of 87.5%, and an area under the curve of 0.95 (95% CI, 0.89-1.00; *P* < .001). This cutoff corresponds to a ratio of coronal/sagittal correction of 0.8:1.

### Postoperative Follow-up and Complications

Mean follow-up was 17.5 ± 9.3 months (range, 5-35). Planned subsequent ACL revision (n = 7) was carried out after 9.0 ± 7.1 months (range, 4-26). Removal of the plate occurred in 13 cases at 15.4 ± 7.6 months (range, 7-31) after the initial operation due to irritating hardware. Complications occurred in 2 patients. In 1 patient, a wound-healing disorder occurred with consequent revision surgery including plate exchange and antibiotic treatment for 8 weeks. One patient had a loose body in the knee joint, which was arthroscopically removed 7 months after the initial operation.

## Discussion

To the best of our knowledge, this is the first study to investigate the 3D accuracy of HTSCO and to analyze the influence of an OWO, a CWO, and the HAA on the accuracy of the PTS correction. The major finding of the study is that HTSCO can be achieved accurately using PSI. Additionally, CWO demonstrated increased accuracy as compared with OWO, which strongly depended on the HAA.

The major finding of the study is that high accuracy could be confirmed in navigated HTSCO with the used PSI system. Brinkman et al^3^ summarized that one of the leading negative prognostic factors after HTO is under- or overcorrection. Therefore, achievement of the highest possible accuracy in such procedures should be the leading principle. A systematic review^44^ on accuracy of conventional HTO showed that in 8 of 14 cohorts, <75% of the patients were in an acceptable range of accuracy postoperatively. It was subsequently demonstrated that PSI-navigated HTOs are much more accurate,^10,31,39^ with a range of accuracy of −0.1° to 0.2° in the coronal plane and −0.1° to 1.3° in the sagittal plane. As opposed to these studies, we used absolute values for calculation of the accuracy, which inevitably led to larger values (1.9°± 1.4° for coronal plane; 2.3°± 1.1° for sagittal plane). In the future, within the context of accuracy measurement, it is advisable to consistently use absolute values because inaccuracies in both directions (+/−) should be avoided. Furthermore, the use of relative numbers can lead to a false high level of accuracy.

An additional finding of the study is that CWO was significantly more accurate in PTS correction than OWO for HTSCO. Because no other study compared 3D accuracy differences between CWO and OWO, not even for classic HTO, no comparison with the literature can be made. However, a meta-analysis of classic HTO without PSI^5^ found that open wedge HTO may accidentally increase posterior slope, and closed wedge HTO may accidentally lead to decreased posterior slope. This is in line with our findings that the CWO is likely inherently more advantageous for reducing PTS.

A further finding of the study is that the error in sagittal correction correlated to an increasing HAA. We identified the HAA cutoff at 38.9° for a sagittal accuracy of <2° in HTSCO. A reason for the worse accuracy of OWO with increasing HAA could be the greater technical and anatomic challenges for OWO as HAA rises.^11^ For extreme HAA, the anteromedial approach to the tibial head does not provide an orthogonal view to the cutting and spreading plane. Therefore, the cutting and spreading devices cannot be used orthogonally to the hinge axis. Furthermore, the distance of the hinge axis to the proximal tibiofibular joint lengthens with increased HAA (Figure 5B) as well as the thickness of the cortical bone changes,^35,38^ with both leading to a less stable hinge.

We suggest that a CWO should be considered for HTSCO with the primary goal of PTS correction. While our study does not provide clear evidence that an OWO should be considered for HTSCO with the primary goal of coronal correction, this assumption can be made. To investigate this matter, additional cases with HAA values between 65° and 90° need to be conducted and analyzed for a more robust conclusion. In recognition of the importance of the difference between CWO and OWO, additional combined accuracy and clinical studies are needed with large patient populations and long-term follow-up focusing on the difference between the CWO and OWO techniques.

A limitation of the current study is the small sample size, although it is important to acknowledge that the patient population is comparable to or even larger than that of other studies examining the accuracy of PSI in HTO.^14,39,45^ Second, the loss of 6 patients owing to the lack of postoperative CT may increase the risk of selection bias. Third, incorporating additional clinical scores would complement the study. However, as our primary focus was on accuracy, the clinical score would not add information to answer our hypothesis. Fourth, the preference of planning CWO over OWO when the main correction was in the sagittal plane (slope) rather than in the coronal may lead to a selection bias, and the heterogeneity in the indications for surgery may have introduced confounding factors. Finally, it is worth noting that a control group undergoing conventional HTSCO would be valuable to validate the benefits of the presented PSI system. However, our decision to maximize the inclusion of cases in the PSI group was driven by the aim of understanding the potential advantages and risks associated with this new technology as thoroughly as possible.

## Conclusion

Slope-reducing osteotomy can accurately be achieved using PSI. CWO demonstrated increased accuracy as compared with OWO, which strongly depended on the HAA.

With an aim of combined PTS and coronal correction, CWO should be considered the primary choice for accurate slope reduction with a coronal/sagittal correction cutoff of 0.8:1 (HAA = 38.9°).
